# Numerical Simulation on Pulsed Laser Ablation of the Single-Crystal Superalloy Considering Material Moving Front and Effect of Comprehensive Heat Dissipation

**DOI:** 10.3390/mi12020225

**Published:** 2021-02-23

**Authors:** Bin Wang, Yihui Huang, Junke Jiao, Hao Wang, Ji Wang, Wenwu Zhang, Liyuan Sheng

**Affiliations:** 1 Institute of Advanced Manufacturing Technology, Ningbo Institute of Materials Technology and Engineering, University of Chinese Academy of Sciences, Ningbo 315201, China; wangbin@nimte.ac.cn (B.W.); jiaojunke@nimte.ac.cn (J.J.); wji@nimte.ac.cn (J.W.); zhangwenwu@nimte.ac.cn (W.Z.); 2 College of Materials Science and Opto-Electronic Technology, University of Chinese Academy of Sciences, Beijing 100049, China; 3 Chair of Applied Laser Technologies, Ruhr-Universität Bochum, 44801 Bochum, Germany; Hao.Wang-p6i@ruhr-uni-bochum.de; 4 Labortary of Advanced Materials and Processing, PKU-HKUST ShenZhen-HongKong Institution, Shenzhen 518057, China

**Keywords:** pulsed laser ablation, numerical simulation, material moving front, heat dissipation, DD6 single-crystal superalloy

## Abstract

In the present research, an iterative numerical model is proposed to investigate the nanosecond pulsed laser ablation (PLA) mechanism of the DD6 single-crystal superalloy. In the numerical model, two subroutines are introduced to trace the moving boundary and update the thermal load. The iteration between the main governing equation and the two subroutines enables the PLA numerical simulation to consider material moving front and effect of comprehensive heat dissipation including thermal convection and radiation. The basic experimental results exhibit a good agreement with simulation results which indicates the good accuracy of the simulation model. Therefore, the PLA mechanism of the DD6 single-crystal superalloy is studied base on the improved iterative model, which indicates the evolution of temperature field, ablation zone morphology, formation of recast layer and heat-affected zone are closely related with time. The temperature of the laser spot center increases sharply at the first stage, reaching a maximum value of 5252 K, and then decreases gradually. The thermal dissipation postpones the ablation rate but promotes the formation of a recast layer and heat-affected zone. Due to the evaporation and thermal dissipation, the depth of the molten layer exhibits two rapid increasing stages. The comprehensive analysis of the PLA processing by the improved simulation model helps the understanding of the intrinsic mechanism, which would contribute to the further optimizing parameters of PLA fabrication of the DD6 single-crystal superalloy.

## 1. Introduction

Pulsed laser ablation (PLA) techniques have been widely applied to nanomaterial production [1,2], film deposition [3], and material processing [4,5,6,7]. Especially for surface-texture micromachining of some difficult-to-machine materials such as ceramic and superalloy, PLA techniques exhibit more advantages due to their high precision, high flexibility, and low damage [8]. During the past two decades, researchers have investigated the mechanisms of pulse laser ablation of metallic materials [9,10]. The consensus has been reached that the evaporation and phase explosion were the main ablation mechanisms, and the phase explosion only occurred when the laser fluence was high enough that the temperature of the material reached the 0.9 times of the critical temperature [11,12]. Despite the developments of basic ablation mechanism, in-depth understanding of the absorbing, transmitting, and dissipating of laser energy is still limited, owing to the limitations of conventional experimental methods [13]. As a rapidly developed method, the numerical simulation study could reproduce the laser ablation process, which provides an effective method for in-depth analysis of the PLA mechanism. However, it is still difficult to build an ideal analytical model. Therefore, to develop an accurate and high-fidelity numerical model would be very valuable.

During the PLA processing, the continuous surface recession induced by laser ab-lation results in a moving boundary which changes the shape of the solution domain and brings great challenge to numerical modeling [14]. PLA modeling should solve the thermal behaviors of solid materials under pulsed laser irradiation, including ablation, heat conduction, heat convection, and radiation. Different from the traditional thermal problems, the PLA problem involved a moving front [15]. The core problem is how to deal with the coupling between the thermal behaviors and the shape change of the solution domain induced by the moving boundary. In previous research, the decoupling scheme was adopted, the solution to thermal response was assumed to be independent of the boundary recession without considering the ablation-induced shape change of the solution domain [16,17,18]. According to Hertz-Knudsen equation describing the surface evaporation rate, the ablation morphology was estimated by the integration of the evaporation rate over time. As the ablation material was supposed to be removed promptly along with the dissipation of the laser energy and laser-induced heat flux was supposed to be bonded with the moving boundary, then the decoupling scheme would underestimate the ablation depth, the recast layer and heat-affected zone [19].

Wang argued the solution domain and the laser heat flux should be updated sim-ultaneously with the moving boundary progressively, which ensured the laser was always irradiating on the ablation boundary [20]. Based on the ALE adaptive remesh algorithm, Wang proposed a strong coupling model which solved the thermal behaviors and updated the shape change of the target material simultaneously. Results showed that the strong coupling model achieved a better agreement with experimental data, compared with the previous model. Zhang adopted the strong coupling scheme and proposed a model considering the instantaneous material removal under low laser fluence. As the surface temperature is usually as high as several thousands of K during the PLA process, the surface thermal convection and radiation effect are nonignorable, so the model also considered the effect of comprehensive heat dissipation including the thermal convection and radiation effect [21]. Nevertheless, the model adopted a boiling temperature-based element deletion algorithm, the element would be deleted immediately when its temperature reached the boiling point. According to Hertz-Knudsen equation, the vaporized material was removed at a certain rate rather than in a flash when it reaching the boiling point. The boiling temperature-based element deletion algorithm would also lead to some errors in the model. As mentioned above, a strong coupling scheme should solve the thermal response behaviors and update the shape change of the target material simultaneously, which is important for the improvement of the model accuracy. In order to reproduce the PLA process more realistically, the thermal convection and radiation effect should be considered. Moreover, the material removal rate should also follow the Hertz–Knudsen equation. However, till now there is rarely few reports on this kind of model.

The DD6 single-crystal superalloy is the second generation of nickel-based single-crystal superalloy in China and possesses the advantages of good high-temperature mechanical properties, good long-term microstructure stability and good casting properties [22,23,24]. The DD6 single-crystal superalloy could be applied to fabricate high-temperature components such as working blades of gas turbines with complex internal cavities. Actually, the nickel-based superalloy is composed of γ matrix, γ’ strengthening phase, carbides, borides and other phases [25,26,27,28]. The difference of these phases in physical properties would affect the laser ablation. Moreover, laser processing also could exert obvious influence on the processed materials [29,30]. The heat diffusion during the laser processing would result in the recrystallization and phase transformation, which would influence the microstructure stability and mechanical properties [31,32,33,34]. Therefore, how to well control the laser ablation processing with less influence on the matrix is very important for the laser ablation of the single-crystal superalloy. The building of optimal simulation model would contribute to the optimization of the laser ablation.

In the present research, a 2-D axisymmetric numerical model was proposed to in-vestigate the PLA mechanism of the DD6 single-crystal superalloy, combining with some experimental analysis. The model utilized a strong coupling iterative scheme to consider the material moving front, which solved the heat conduction equation and updated the ablation-induced moving boundary and the thermal load conditions simultaneously. Moreover, the effect of comprehensive heat dissipation including thermal convection and radiation was also considered in the model. Besides, considering the material nonlinearity by temperature variation, the temperature-dependent material properties of the DD6 single-crystal superalloy were used in the model, and the simulated results showed good agreement with the experimental results. Therefore, the model was used to investigate the PLA mechanism of the DD6 single-crystal superalloy, and the absorption of laser energy, the evolution of ablation morphology, the development of recast layer and heat-affected zone were analyzed in depth.

## 2. Theoretical Formulation

**Figure 1:**
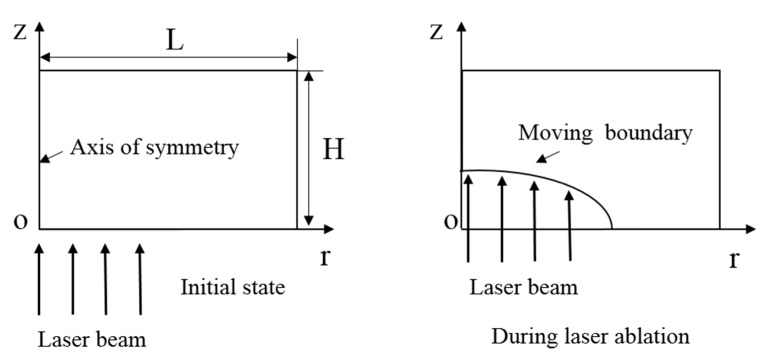
The model description.

The effect of nanosecond laser energy absorption on the target material is heating, and the heat transfer inside the material can be described by the following heat conduction equation [20]:

(1) $${\rho C}_{P}\frac{\partial T}{\partial t}-\nabla \cdot (k\nabla T)=\dot{q}$$

where *ρ*, *C_P_* and *k* are density, specific heat and thermal conductivity of the target material, respectively. *T* and *t* are the temperature and time respectively. An axisymmetric model is proposed here to reduce the computational cost. The *z* axis is the axisymmetric axis where the incident laser beam comes from the *z*-axis positive direction and the *r* axis represents the radial axis. It is worth noting that, during the ablation process, the laser irradiated surface always keeps moving due to the material removal by laser ablation. However, the moving boundary brings great challenge to the numerical simulation study, which will be discussed later. The $\dot{q}$ is the absorbed laser energy which is assumed to the volume heat source, and expressed as:

(2) $$\dot{q}=(1-R_{f})\alpha I_{0}(r,t)e^{-\alpha z’}$$

where *R_f_* is the surface reflectivity and *α* is the absorption coefficient of the material. when *z*’ = 0, the surface represents the moving boundary, and *z*’ is the distance in the *z*-axis direction from any point to the moving boundary. During the PLA process, the laser beam keeps irradiating on the moving boundary, and the heat source induced by laser absorption has been bonded with the moving boundary, as shown in Figure 1. The *I*_0_(*r*, *t*) is the power density of incident laser beam. In general, for the Nd:YAG laser in the current study, the *I*_0_(*r*, *t*) can be expressed as:

(3) $$I_{0}(r,t)=I_{\max}{\left(\frac{t}{t_{\max}}\right)}^{7}\exp(-2\frac{r^{2}}{r_{0}^{2}}+7(1-\frac{t}{t_{\max}}))$$

where *I_max_* is the peak heat flux of the laser pulse; *r*_0_ and *t_max_* are the spot radius and the full width at half maximum (FWHM) of the laser pulse for time profile, respectively. Specifically, the heat flux of the laser pulse reaches to its peak value *I_max_* at the time of *t_max_* for the current study.

The ablation rate *ν_s_* is the rate of the material removal which includes the evaporation and phase explosion. When the incidence laser fluence is sufficiently high, the phase explosion occurs, which leads to a rapid increase in ablation rate [11]. When the incidence laser fluence is relatively lower, the material removal is predominantly induced by the material evaporation. Then the corresponding surface recession rate is equal to the vaporization rate of the material, and the surface recession rate *ν_s_* can be described by the Hertz-Knudsen equation [15]:

(4) $$\nu_{s}=\beta \sqrt{\frac{m}{2\pi k_{B}^{}T_{s}}}\frac{P_{b}}{\rho }\exp(\frac{mL_{\nu }}{k_{B}}(\frac{1}{T_{b}}-\frac{1}{T_{s}}))$$

where *β* and *m* are the vaporization coefficient and atomic mass of the target material, *L_ν_* is the latent heat of vaporization. *T_s_* is the ablation surface temperature of the solid material and *T_b_* is the boiling point at the standard atmospheric pressure *P_b_* (where *P_b_* = 1 atm). The *k_B_* is the Boltzmann constant. Moreover, the initial conditions and boundary conditions are expressed as:

(5) $${T(r,z,t)}_{t=0}=T_{0}^{}$$

(6) $$\frac{\partial T(r,z,t)}{\partial r}|{{}_{r=\frac{L}{2}}=\frac{\partial T(r,z,t)}{\partial z}|}_{z=H}=0$$

where Equation (5) is the initial conditions, *T*_0_ is the initial temperature of target material; Equation (3) is boundary conditions on both the rear surface and lateral surface, which are adiabatic boundaries, as shown in Figure 1. *L* and *H* are the length and width of the solution domain. On the moving boundary, the energy balance is described as follows [21]:

(7) $${k\frac{\partial T}{\partial n}|}_{z’=0}={L_{\nu }\rho \nu_{s}|}_{z’=0}+{h(T_{s}-T_{a})|}_{z’=0}+\varepsilon \sigma {\left(T_{s}-T_{a}\right)}^{4}|z’=0$$

From left to right, each item in Equation (7) represents the thermal conduction, heat loss due to ablation, thermal convection and thermal radiation, respectively. Where *n* is the surface normal unit vector, and *T_a_* is the ambient temperature; *h* is the convective heat transfer coefficient, while *ε* and *σ* are the emissivity of the target material and the Stefan– Boltzmann constant, respectively.

## 3. Numerical Implementation

The coupled equations (Equations (1)–(7)) should be solved simultaneously, while the great challenge for the current study is from progressive material removal induced by laser ablation, which is reflected in two aspects.

(1) The progressive material removal results in a moving boundary, and the shape changing of the solution domain due to the moving boundary is coupled with the heat conduction behavior. Therefore, during the process of modeling, the shape of the solution domain should be updated simultaneously in time with the corresponding surface recession.

(2) As all the thermal load conditions (including laser-induced heat flux, heat loss due to ablation, thermal convection and radiation) have been always bonded with the moving boundary, during the process of modeling they should be updated in time with the corresponding surface recession as well.

In view of the above challenges, a strong coupling numerical iteration method is adopted in the current numerical implementation. Equation (1) as the main governing equation is solved by using finite element method (FEM). In order to overcome the two challenges above, two subroutine modules are established.

### 3.1. Moving Boundary Tracing Subroutine

The first subroutine is the moving boundary tracing subroutine (MTS) which enables us to track the moving boundary and capture the shape change of the solution domain. MTS lays a foundation for the coupling solution between shape change and thermal behavior. Equation (8) describes the surface instant recession rate during PLA, based on which the new *Z*-axis coordinate value of each surface node after each time increment is obtained by:

(8) $$z^{{}_{n+1}}=z^{{}_{n}}+\nu_{s}^{n}\Delta t$$

where *n* is the time increment number (*n* = 0, 1, 2, 3……) and *ν_s_^n^* is the ablation rate at the time nΔt obtained from Equation (8), while Δt is the time step for every iteration. A deformation mesh method combined with an adaptive remesh algorithm is adopted here to regenerate the new mesh after every time-step increment to be consistent with the shape change. The adaptive remesh algorithm enables us to smooth the mesh and control the mesh distortion. When the ablation depth *d* exceeds the half of the thickness of the target material domain, the mesh deformation becomes much larger. In that case, severe mesh distortion arises with the absence of the remesh algorithm. Therefore, it is quite necessary to involve a remesh algorithm, which adjusts the mesh shape in time by evaluating the mesh quality [20]. It would make sure that the mesh quality is always in a good state during the modeling process. To avoid severe mesh distortion, the following two factors should be paid more attention. Firstly, the thickness of the target material domain should to be much larger, compared to the expected ablation depth. In this way, the mesh deformation induced by the surface instant recession will gain more space to release by averaging the mesh deformation to the whole solution domain. Secondly, as the update frequency of mesh deformation is closely related to the time step, a sufficiently smaller time step Δt should be taken. When the time step becomes much smaller, the mesh update becomes more frequent, which can effectively avoid mesh distortion. Moreover, the smaller time step could also improve the numerical modeling accuracy. For the modeling case of laser ablation with a constant ablation rate of 1 mm/s and a duration of 1 s, the results showed that the percent error is 97.4% when the time step is Δt= 1 s. If the time step decreases to Δt= 0.001 s, the percent error would drop to 2.7% [31]. However, too small a time step will lead to a lot of time consumption, increasing the calculation cost. To balance the accuracy and the calculation cost, the time step should be chosen reasonably.

### 3.2. Thermal Load Updating Subroutine

The second subroutine is the thermal load updating subroutine (TUS) which enables us to accurately update the thermal load distribution in time, including laser-induced surface heat flux, the heat loss due to vaporization, thermal convection and radiation. As described in Equation (7), all the thermal loads are bonded with ablation moving front boundary (where *z*′(*r*) = 0). As the first subroutine (MTS) enables us to precisely track the moving front boundary, and make an accurate transfer of position coordinate of the moving front boundary to TUS module, the TUS module will update all the thermal loads described in Equation (7) in time according to the position coordinate data from MTS at each time step increment.

### 3.3. Numerical Solution Flow

A strong coupling numerical iteration algorithm between the solution of heat conduction equation (Equation (1)) and the above two subroutines (MTS, TUS) is adopted in present study. The heat conduction equation will be solved by using the finite element method (FEM). MTS subroutine implementation enables us to track the moving front and capture the shape change of the solution domain, and TUS subroutine implementation enables us to accurately update the variation of thermal load distribution in time. The iteration achieves the strong coupling of the above coupled equations (Equations (1)–(7)).

In a small time-step, the solution of heat conduction equation is implemented by utilizing the FEM method to obtain the temperature field of the solution domain and the temperature distribution on the ablation moving boundary. Then, the temperature distribution data will be transferred to MTS subroutine. Based on the data, the implementation of MTS subroutine will track the moving front, capture and the remesh the solution domain through the utilization of deformation mesh and adaptive mesh technique. Then MTS subroutine transfers the position data of the moving front boundary to TUS subroutine for the updating of the thermal load. After that the first iteration is finished. Nevertheless, more iterations are needed to control the residual in a reasonable range and obtain more accurate results. Therefore, after the solution domain is remeshed and all thermal loads are updated, the solution of heat conduction equation will be implemented once again, and a new iteration starts. The iterations will not stop until a reasonable range of convergence residual is achieved, then the results in the small time step are convergent, and the numerical implementation in the time-step is achieved. Then the numerical implementation will move to next time-step, the iteration is repeated until the whole implementation is finished.

### 3.4. Material Properties and Experimental Parameters

The computational domain with a length *L* of 100 μm and a width *H* of 120 μm is used here. The domain is large enough to cover all the heat-affected zone that the adiabatic boundary assumption on both bottom surface and side surface shown in Equation (3) is reasonable. The gradient mesh size is used here to save the computing resources, and mesh size for the first laser is 0.2 μm which would provide sufficient accuracy in the current simulation study. The chemical composition of the DD6 single-crystal superalloy is listed in Table 1, and the material properties of DD6 nickel-based superalloy are shown in Table 2 and Table 3, which are obtained from the Aviation Materials Manual and the ref [20,21]. The temperature-dependent material properties are included in the simulation study to obtain a more accurate result. Laser frequency in the experiment is set to 5000 Hz, and the period between each pulse is 200 μs. The simulation time is set to 200 μs to investigate the ablation behavior in one laser pulse period. The wavelength and pulse width of the laser are 532 nm and 60 ns, respectively, with the spot diameter of 60 μm.

## 4. Results and Discussion

### 4.1. Effect of Laser Fluence and Model Validation

**Figure 2:**
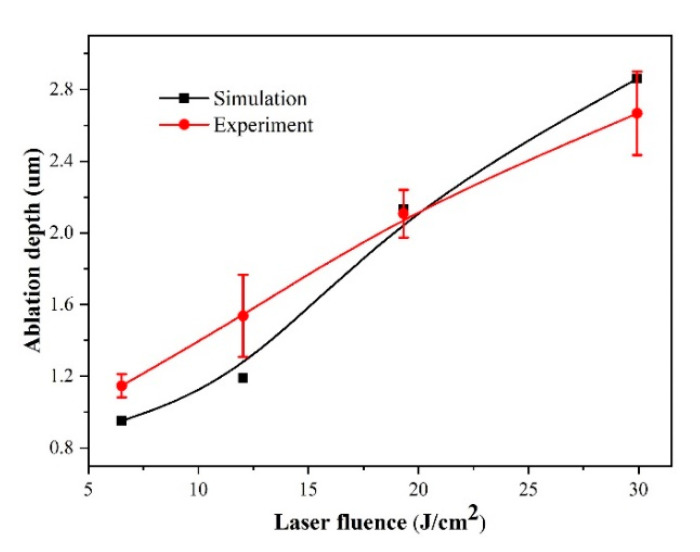
Comparison of ablation depth between experiment and simulation results under different laser fluence.

The experimental study on the effect of laser fluence was performed and repeated three times, as shown in Figure 2. The ablation depth was evaluated by using the laser confocal microscope, and the ablation depth represents the value of the spot center. It was found that the ablation depth increased with the laser fluence in the experiment. They almost have a linear relationship. Based on the experiment results, it can be deduced that the phase explosion and plasma shielding is unlikely to occur because of the low ablation depth. The simulation results demonstrate that the ablation depth also increases with the increasing of laser fluence. The simulation results have a good agreement with the experiment results, which indicates the accuracy of the simulation model is relatively good.

### 4.2. Ablation Process

**Figure 3:**
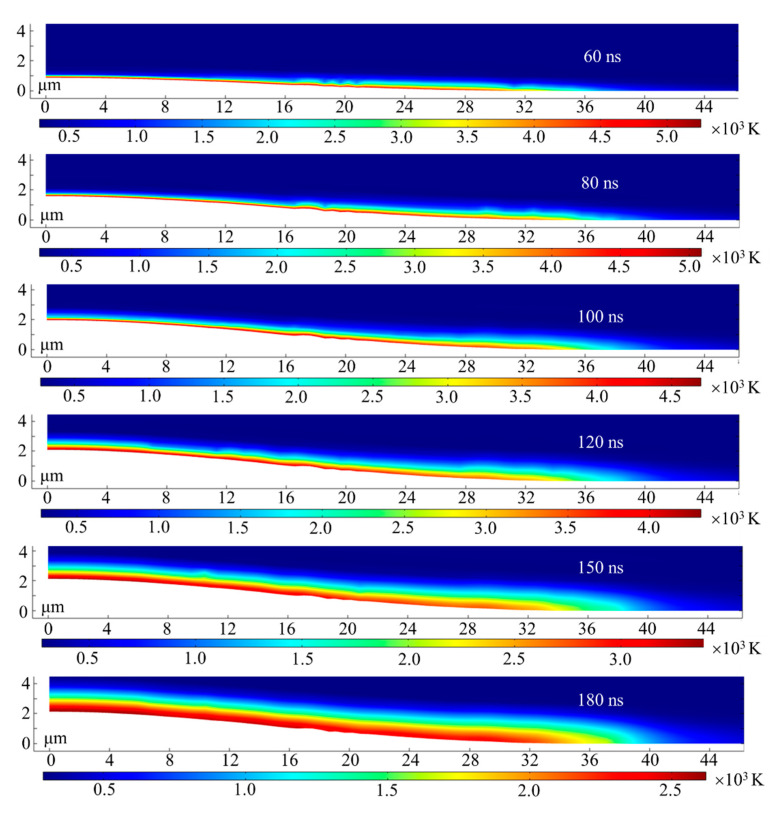
Contour plots of ablation morphology and temperature for the simulated ablation for laser fluence of 19.3 J/cm^2^.

The ablation process for the laser fluence of 19.3 J/cm^2^ is discussed here as a typical example. Equation (3) shows the temporal distribution of incident laser energy is in a near-Gaussian distribution and most energy is absorbed in the laser duration of 2 × *t_max_* (120 ns). Figure 3 shows the contour plots of the temperature and ablation morphology at different time. During early stage of the laser irradiation (when *t* < 100 ns), only a thin layer of the material near the surface is heated to several thousand K. That should be ascribed to the rapidly vaporization of material which takes away a large amount of heat. At this stage, evaporation is the dominant way of heat dissipation, and very little laser energy is transmitted to the inside material by the means of thermal conduction, so only the material near the surface is heated. It is worth noting that the intensity of laser fluence decreases gradually at the end of laser pulse (when 100 ns < *t* < 120 ns), which leads to the decrease of evaporation rate. After then, only part of the heat is dissipated by evaporation, the remaining heat is transferred to the material in the form of thermal conduction. Therefore, at the end of the laser pulse, the heat-affected zone increases at 120 ns, compared with that at 60 ns, 80 ns and 100 ns. As Equation (3) shows the spatial distribution of incident laser energy with a Gaussian distribution, the intensity of laser fluence decreases gradually from the center to the edge of the spot. For the same reason the heat-affected zone near the edge region of laser spot is thicker than that in the spot central region. When laser irradiation stops (when *t* > 120 ns), the surface temperature drops below the boiling point rapidly due to the absence of laser radiation, the heat dissipation of thermal conduction, thermal convection and radiation. Moreover, the surface evaporation of the material will stop immediately. At this stage, the dominant energy dissipation form includes the thermal conduction, thermal convection and radiation, and the heat-affected zone will increase rapidly due to the thermal conduction (*t* = 150 ns and 180 ns). The formation of the heat-affected zone and recast layer could be mainly ascribed to the part of heat transferred into the material in the form of thermal conduction. Therefore, it is necessary to analyze the effect of thermal conduction deeply.

**Figure 4:**
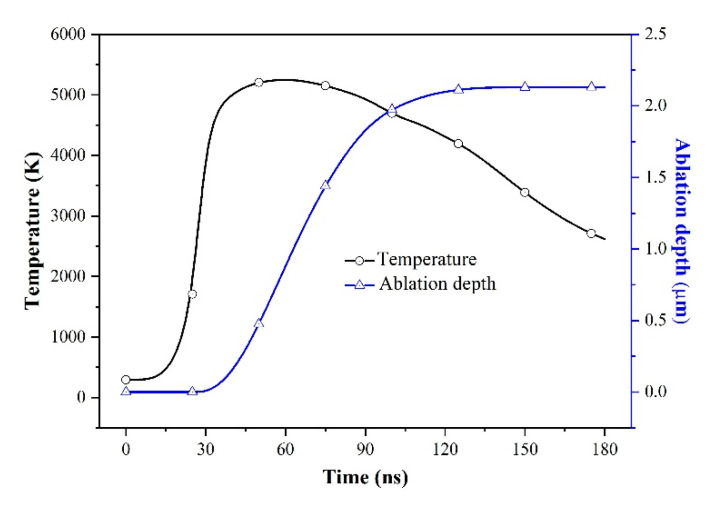
Variation of simulated temperature and ablation depth of laser spot center with time for laser fluence of 19.3 J/cm^2^.

The temperature and ablation depth evolution of laser spot center is shown in Figure 4. At 30 ns the surface temperature reached the boiling point (3005 K) and then the material begins to vaporize. At the early stage of ablation (when 30 ns < *t* < 45 ns) the temperature at laser spot center keeps increasing rapidly and then the increasing rate decreases gradually (40 ns < *t* < 60 ns). That could be attributed to the increased vaporization. When 30 ns < *t* < 45 ns, the surface temperature is relatively lower, the vaporization rate *ν_s_* and surface temperature increase rapidly. Due to less heat dissipation for vaporization, the surface temperature at this stage increases rapidly. When 45 ns < *t* < 60 ns, the surface temperature reaches a high level but the vaporization rate *ν_s_* is still in rapid increasing stage. Because the heat dissipation is mainly used for the evaporation, the surface temperature almost keeps little increase. As the surface temperature is comprehensively affected by the incident laser intensity, ablation rate, thermal conduction, thermal convection and radiation, so the present result is the competition of all the factors. The temperature also reaches the maximum 5252 K at 60 ns, which indicates the intensity of laser fluence reaches the maximum. After that, the surface temperature decreases due to the decrease of the laser intensity. The ablation depth continued to increase rapidly until the surface temperature drops to near the boiling point at about 130 ns.

**Figure 5:**
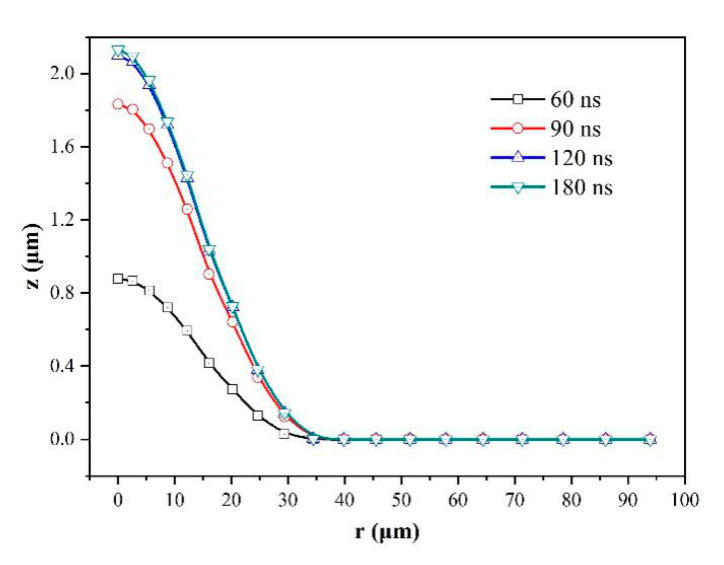
Variation of simulated ablation morphology with time for laser fluence of 19.3 J/cm^2^.

**Figure 6:**
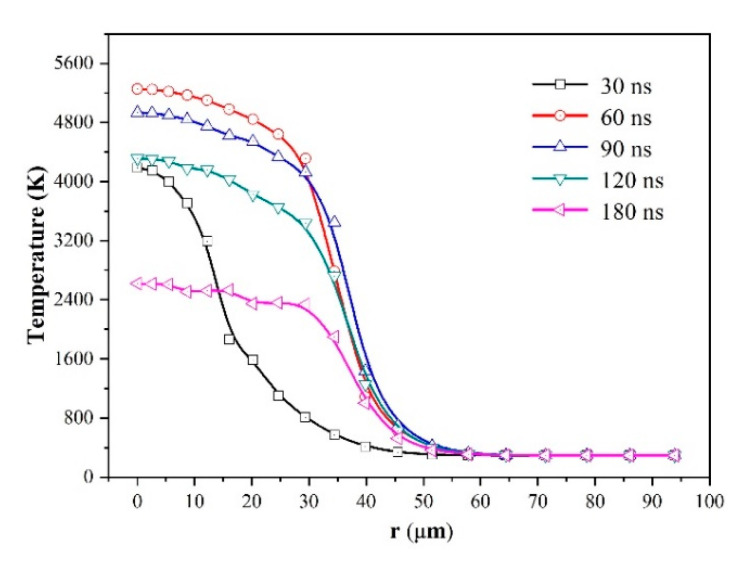
Variation of simulated surface temperature distribution with time for laser fluence of 19.3 J/cm^2^.

The variations of ablation morphology and surface temperature with time are shown in Figure 5 and Figure 6, respectively. The results further confirmed that the ablation almost stopped at the end of the pulse. It is interesting that the ablation morphologies of 120 ns and 180 ns are almost coincident. At the early stage (when *t* = 30 ns), the temperature of spot center reaches to over 4000 K, but near the spot edge region the temperature is lower. When *t* > 30 ns, the temperature in the laser spot region became more uniform, because of the ablation heat dissipation and thermal conduction effect.

### 4.3. Recast Layer and Heat-Affected Zone

Since the laser frequency is 5000 Hz and the time interval between two pulses is 200 μs, the simulation time is set to 200 μs in order to investigate the thermal behavior in one pulse period. The duration of the laser pulse is only 120 ns, which indicates the material with short-period laser heating will go through a relatively long cooling stage before the next laser pulse. During the cooling stage, the heat will be dissipated in the form of thermal conduction, convection and radiation, which decreases the temperature of material gradually. Owing to the influence of thermal conduction, the recast layer and heat-affected zone would form inside the material.

**Figure 7:**
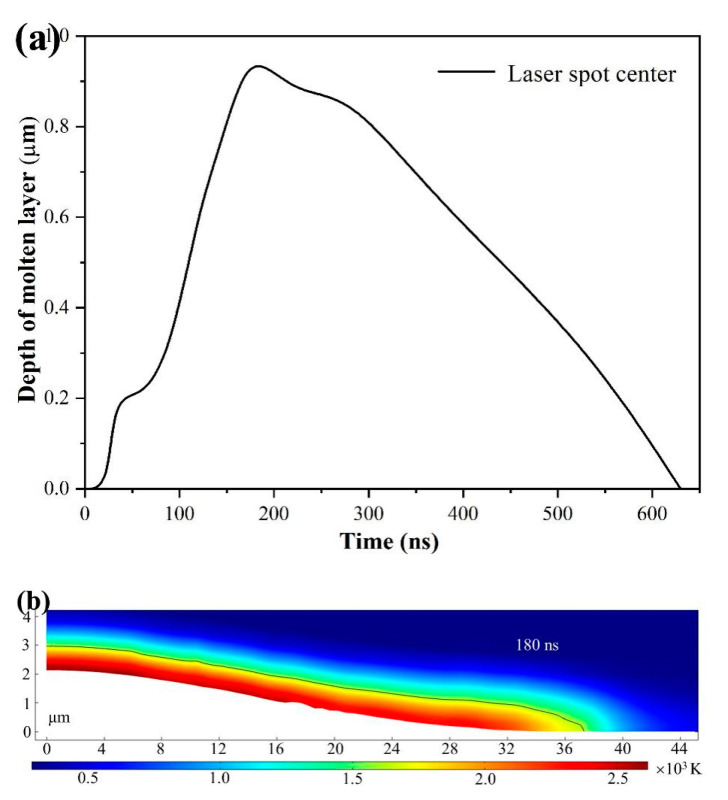
Evolution of simulated molten laser depth for laser spot center with time for laser fluence of 19.3 J/cm^2^ (**a**) and the corresponding recast layer plot at 180 ns (**b**).

The evolutions of molten layer depth for laser spot center with time and the corresponding recast layer plot at 180 μs are shown in Figure 7. The surface temperature reaches to the melting point at 25 ns and the material begins to melt, as shown in Figure 7a. At 30 ns, the surface temperature reaches the boiling point, and the material begins to evaporate. After then, a stage appears with low increasing rate of molten layer depth, because of the heat dissipation of evaporation. When the heat dissipation decreases (*t* > 60 ns), the heat conducted to the interior material increases, and the molten layer depth would increase rapidly again. The molten layer depth keeps increasing even when the laser irradiating stops (*t* > 120 ns), which should be attributed to the much higher surface temperature. The redundant heat is thermally conducted from the surface to inside and induces the melting of interior material. The molten layer depth reaches its maximum (0.96 μm) value at 180 ns, and then the molten material is gradually cooled and solidified to form a recast layer. At 630 ns, all molten material solidifies into solid material and forms the recast layer. As shown in Figure 7b, the morphology of recast layer indicated by the black line changes with the distance, and the recast layer depth for laser spot center is 0.96 μm.

**Figure 8:**
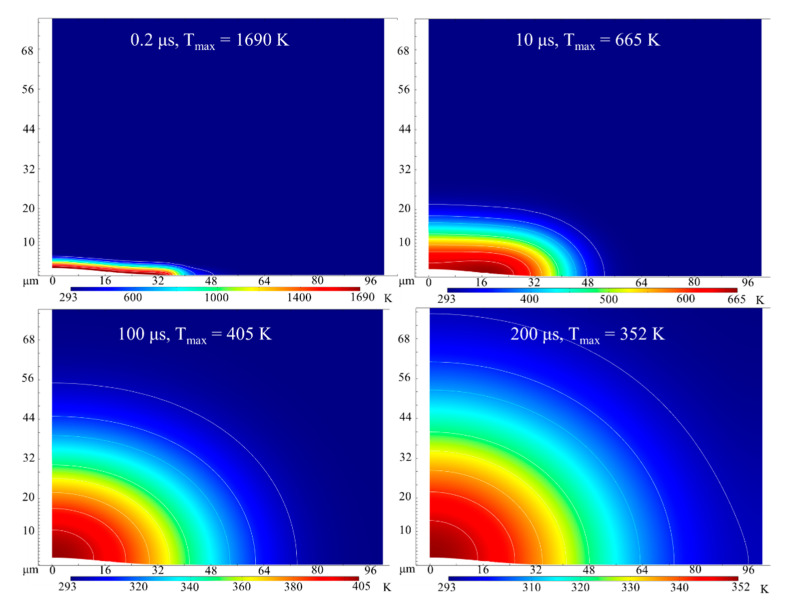
Evolution of simulated temperature distribution and heat-affected zone with time for laser fluence of 19.3 J/cm^2^.

After the laser heating, the laser heated material is cooled for about 200 μs before the next laser pulse. The evolution of temperature distribution and heat-affected zone with time are shown in Figure 8. At 0.2 μs, the maximum temperature of *T_max_* is 1690 K, and the heat-affected zone is relatively small. Owing to the heat dissipation of thermal conduction, convection and radiation, the material is cooled down slowly in a relatively long time. The heat-affected zone keeps growing and the temperature gradually decreases to the room temperature. At 200 μs, the maximum temperature *T_max_* decreases to 352 K, and the depth of heat-affected zone is about 75 μm (The depth of heat-affected zone represents the maximum depth of the zone with temperature rise).

Based on the simulation above, it can be found that the ablation of the DD6 single-crystal superalloy begins after the temperature exceeding the boiling temperature, which indicates the happening of evaporation. When the temperature is above 5000 K, the ablation rate becomes the highest and almost keeps constant. When the temperature is below 5000 K, the ablation rate decreases greatly. That indicates the phase explosion would play an important role when the temperature is above 5000 K. Such a deduction also can be confirmed by the surface temperature and ablation morphology. The central region with radius about 10 μm has the highest temperature and the deepest ablation morphology. With the radius extending further, the decrease rate of the ablation morphology is more than that of the temperature, which means the decrease of phase explosion. Therefore, it can be concluded that the evaporation works in the whole ablation of the DD6 single-crystal superalloy but the phase explosion plays the main role. Due to the thermal diffusion, the matrix adjacent to the ablated region could be affected. And the thickness of the heat-affected zone changes a little with the depth of the ablation morphology and time, which could be ascribed to the dissipation of energy [35,36]. The main heat-affected zone has the thickness about 1 μm. However, the present study mainly focuses on the single-pulse laser ablation behavior, which is helpful to study the multipulse ablation behavior. It would be more reasonable to investigate the multipulse behavior by way of analogy based on in-depth understanding of the single-pulse ablation mechanism, which will be investigated in the future research.

## 5. Summary

In the present research, an iterative numerical model is proposed to investigate the nanosecond pulsed laser ablation (PLA) mechanism of the DD6 single-crystal superalloy. In the numerical model, two subroutines are introduced to trace the moving boundary and update the thermal load. The iteration between the main governing equation and the two subroutines enables the PLA numerical simulation to consider the material moving front and effect of comprehensive heat dissipation including thermal convection and radiation. The basic experimental results exhibit a good agreement with simulation results which indicates good accuracy of the simulation model. Therefore, the PLA mechanism of the DD6 single-crystal superalloy is studied based on the improved iterative model, which indicates the evolution of temperature field, ablation zone morphology, formation of recast layer and heat-affected zone are closely related with the time. The temperature of laser spot center increases sharply at first stage reaching maximum value of 5252 K and then decreases gradually. The thermal dissipation postpones the ablation rate but promotes the formation of the recast layer and heat-affected zone. Due to the evaporation and thermal dissipation, the depth of molten layer exhibits two rapid increasing stages. The comprehensive analysis of the PLA processing by the improved simulation model helps the understanding of the intrinsic mechanism, which would contribute to the further optimizing parameters of PLA fabrication of the DD6 single-crystal superalloy.

## Figures and Tables

**Table 1:** Chemical composition of the DD6 single-crystal superalloy.

Cr	Co	Mo	W	Re	Hf	Ta	Al	C	Ni
4.3	9.0	2.0	8.0	2.0	0.1	7.5	5.6	0.01	Balance

**Table 2:** Temperature-dependent material properties of the DD6 single-crystal superalloy.

Temperature (K)	Specific Heat (J/kg/K)	Thermal Conductivity (W/m/K)	Reflectivity
373	385	8.00	0.8
573	427	11.15	/
973	566	20.20	0.772
1173	635	24.55	0.4
≥1573	733	33.20	0.4

**Table 3:** Material properties of the DD6 single-crystal superalloy.

Density (kg/m^3^)	Melting Point (K)	Boiling Point (K)	Evaporation Coefficient	Latent Heat of Evaporation (J/kg)	Latent Heat of Melting (J/kg)
8780	1615	3005	1	6.423 × 10^6^	2.96 × 10^5^

## Data Availability

Data available upon request from the corresponding author.
